# Atorvastatin Treatment for Carotid Intima-Media Thickness in Chinese Patients With Type 2 Diabetes: A Meta-Analysis

**DOI:** 10.1097/MD.0000000000001920

**Published:** 2015-11-06

**Authors:** Na Fang, Wei Han, Dandan Gong, Zou Chen, Yu Fan

**Affiliations:** From the Institute of Molecular Biology and Translational Medicine, the Affiliated People's Hospital, Jiangsu University, Zhenjiang, Jiangsu, PR China (NF, DG, ZC, YF); and Department of Pharmacy, Shenyang Children's Hospital, Shenyang, PR China (WH).

## Abstract

Impact of atorvastatin on carotid intima-media thickness (CIMT) in patients with type 2 diabetes is still debating.

The aim of our study is to investigate atorvastatin as adjuvant treatment on CIMT in Chinese patients with type 2 diabetes by conducting a meta-analysis based on the randomized controlled trials (RCTs).

A systematic search of electronic database of the Pubmed, EMBASE, Cochrane Library, VIP database, China National Knowledge Infrastructure, and Wangfang up to January 2015 was conducted. Randomized controlled trials (RCTs) comparing atorvastatin adjuvant treatment to the hypoglycemic therapies or high-dose atorvastatin versus low-dose atorvastatin therapies for patients with type 2 diabetes were selected.

A total of 14 RCTs involving 1345 patients were included. Adjuvant treatment with atorvastatin was associated with a significant reduction in CIMT (weighted mean difference [WMD] = −0.17 mm; 95% confidence interval [CI] −0.22 to −0.12). Compared with the low-dose atorvastatin, high-dose atorvastatin treatment was associated with a significant reduction in CIMT (WMD = −0.17 mm; 95% CI: −0.32 to −0.02). Adjuvant treatment with atorvastatin reduced serum total cholesterol, triglyceride, low-density lipoproteins, and high sensitivity C-reactive protein levels. However, atorvastatin had no significant impact on blood glucose levels.

This meta-analysis demonstrated that treatment with atorvastatin significantly reduced CIMT in Chinese patients with type 2 diabetes. Moreover, high-dose atorvastatin appeared to have additional benefits in reducing CIMT than the low-dose atorvastatin.

## INTRODUCTION

Diabetes mellitus is a global health problem. It is estimated that type 2 diabetes affects at least 285 million people worldwide and the total number of people will rise to 438 million in 2030.^1^ In China, an epidemic study indicated that there were 92.4 million diabetes adults in 2010.^2^ Diabetic patients have at least a 2-fold greater risk of developing cardiovascular disease (CVD) than the general population.^3^ The high mortality and morbidity of patients with type 2 diabetes is mainly because of its vascular complications. Atherosclerosis is the main pathological feature of type 2 diabetic macrovascular complications. Therefore, early management of subclinical atherosclerosis is necessary to prevent serious diabetic complications.

Carotid intima-media thickness (CIMT) is a well-known surrogate marker of subclinical atherosclerosis^4^ and CVD.^5^ Carotid intima-media thickness scanning is a safe, noninvasive, and relatively inexpensive method of assessing subclinical atherosclerosis.^6^ Detection and intervention of CIMT in diabetic patients allows timely treatment and prevention of diabetic vascular complications. Statins therapy has been demonstrated to decrease in the CIMT value, but the drug-specific effects of statins on CIMT are conflicting.^7^ Atorvastatin, is a well-accepted 3-hydroxy-3-methyl-glutaryl coenzyme A reductase drug for management of dyslipidemia in patients with CVD. The role of atorvastatin in CIMT progression has been established in a previously published meta-analysis.^8^However, the impact of atorvastatin on CIMT in patients with type 2 diabetes is still debating.^9^ Therefore, we conducted this meta-analysis based on the available randomized controlled trial (RCT) to provide a comprehensive summary of atorvastatin on CIMT progression in Chinese patients with type 2 diabetes.

## METHODS

### Search Strategies

We systematically conducted a search through PubMed, EMBASE, Cochrane Library, China National Knowledge Infrastructure, Wanfang, and VIP database up to January 2015. The following medical subject headings [Mesh] were used to identify studies: carotid artery intima-media thickness ([Mesh] OR CIMT [Mesh] OR carotid atherosclerosis [Mesh]) AND atorvastatin [Mesh] AND diabetes [Mesh] AND (random [Free Item] OR randomized controlled trials [Free Item] OR RCTs [Free Item]). We also hand searched reference lists of the retrieved papers to identify the additional eligible studies.

### Study Selection

Inclusion criteria were as follows: (1) RCTs investigating atorvastatin treatment or comparison of high-dose versus low-dose atorvastatin in Chinese patients; (2) use of the ultrasound method to measure CIMT at baseline and at end of treatment; and (3) participants were diagnosed of type 2 diabetes based on the diagnostic criteria. High-dose atorvastatin treatment is defined at least 2 times bigger than the low-dose. Carotid intima-media thickness is defined as the measured distance between the luminal-intimal interface and the media-adventitial interface of the common carotid artery.^10^ Trials were excluded if: (1) trial did not evaluate CIMT change as an endpoint; (2) different therapeutic approaches had been used apart from atorvastatin intervention between 2 groups; and (3) nonrandomized controlled trials, case-control study, or cohort study. Patients who had already received atorvastatin or other statins therapy within 2 weeks were excluded.

### Data Extraction and Quality Assessment

Two authors (NF and WH) extracted the following data from eligible articles independently: name of the first author, year of publication, sample size, age, gender, atorvastatin dose/control group dose, duration of treatment, CIMT at baseline, and at end of treatment. The quality of the individual study was evaluated according to the methodological quality of included RCTs using Cochrane risk of bias tool. This tool is based on the following items: random sequence generation, allocation concealment, blinding of participants and personnel, blinding of outcome data, incomplete outcome data, and selective reporting and others.

### Data Synthesis and Analysis

Carotid intima-media thickness was calculated as the weighted mean difference (WMD) or standardized mean difference (SMD) with 95% confidence interval (CI). Analyses were stratified by the atorvastatin versus control or high-dose versus low-dose atorvastatin. Statistical homogeneity was tested using Cochrane's *Q* test and *I*^2^ statistics. *I*^2^ statistic > 50% or *P* < 0.10 in Cochrane's *Q* test was deemed to have significant heterogeneity.^11^ Pooled effect sizes were calculated with a random effects model in the presence of significant heterogeneity; otherwise, a fixed-effect model was selected. Publication bias was determined by using the funnel plot, Begg’ s rank correlation test,^12^ and Egger's regression test.^13^ All the statistical analyses were performed by using STATA statistical software version 11.0 (StataCorp, College Station, TX).

## RESULTS

### Study Characteristics

**FIGURE 1:**
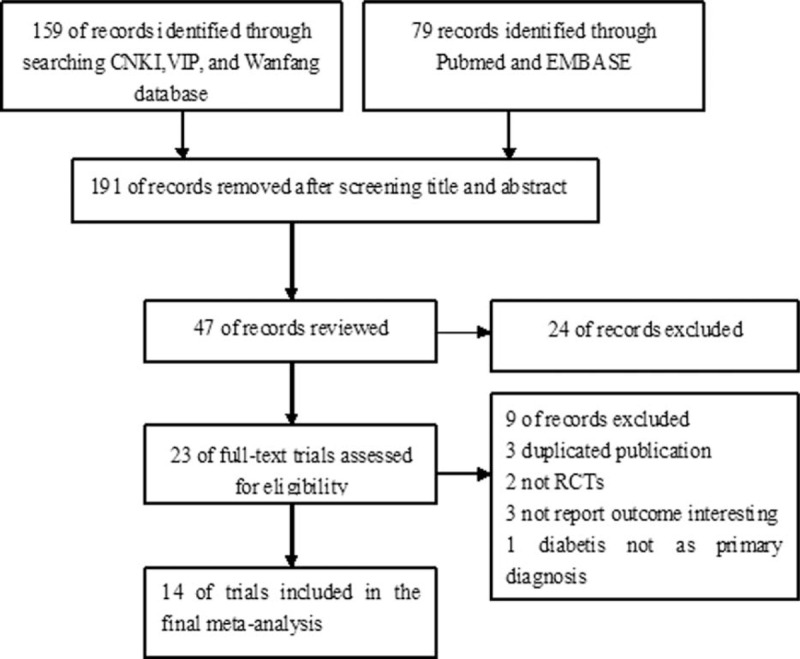
Flow diagram of study selection process.

**FIGURE 2:**
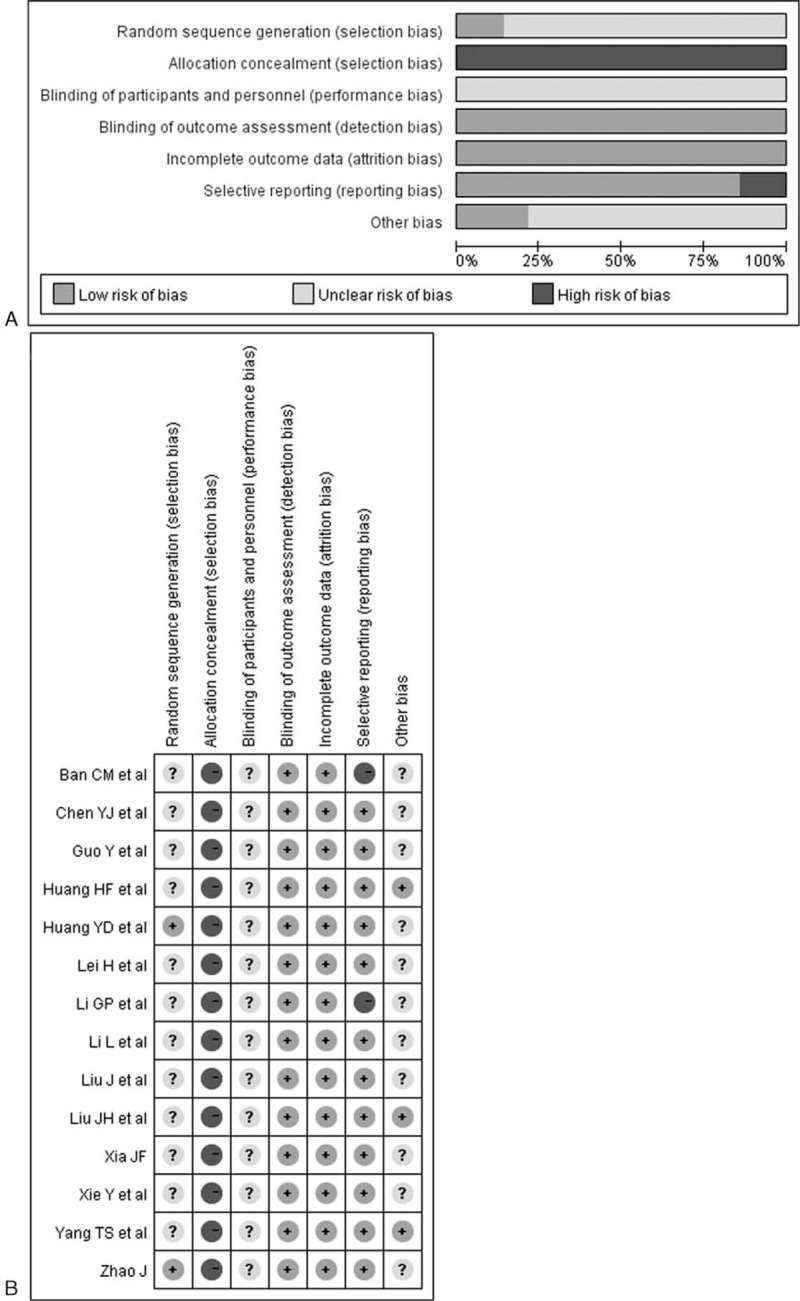
Quality assessment of the included studies. Risk of bias graph (A); risk of bias summary (B).

The primary search yielded 238 records. After applying the predefined inclusion criteria, 14 RCTs^14–27^ were ultimately included in this meta-analysis. The process of selection of the trials is shown in Figure 1. The baseline characteristics of the eligible trials are presented in Table 1 . Of 14 RCTs, 10 trials^14–23^ compared the add-on effect of atorvastatin, and 4 trials^24–27^ provided the comparison of high-dose versus low-dose atorvastatin. A total of 1345 diabetic patients were identified. There was no significant difference in baseline CIMT between 2 groups. The duration of follow-up ranged from 24 weeks to 12 months. Sample size varied from 50 to 155 in the individual trials. The dose of atorvastatin was 10 mg, 20 mg, and 40 mg per day. Carotid intima-media thickness was measured by high-resolution B-mode carotid ultrasonography. In general, the included trials were in moderate quality and the detail quality assessments are shown in Figure 2.

**TABLE 1:**
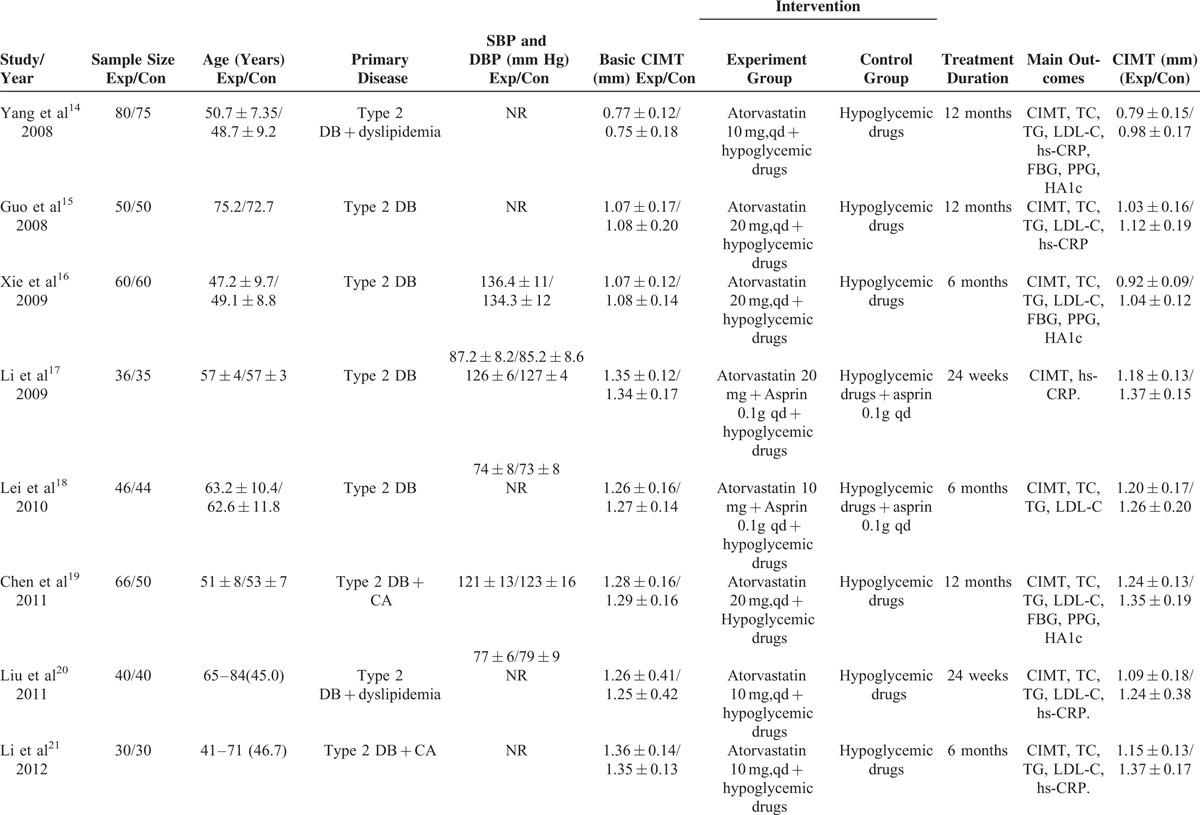
Summary of the Characteristics of the Included Studies

**TABLE 1 (Continued):**
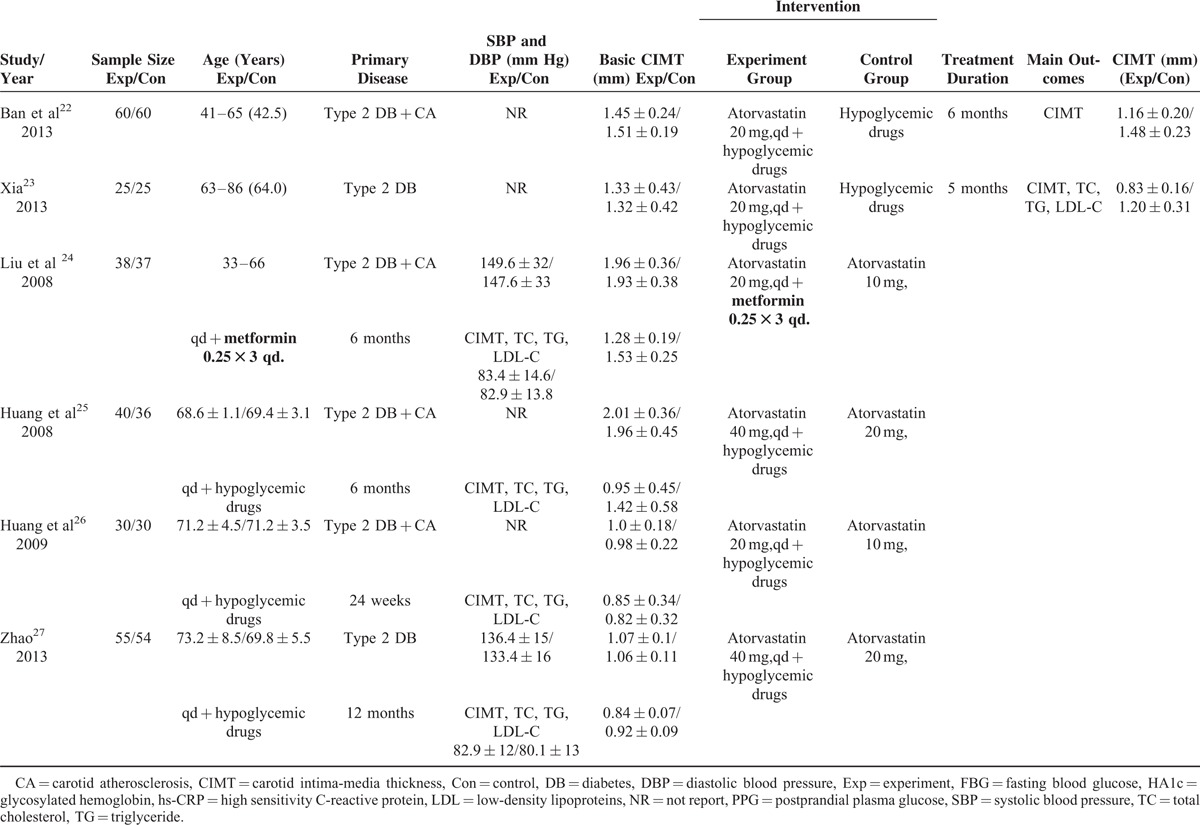
Summary of the Characteristics of the Included Studies

### Changes of CIMT

**FIGURE 3:**
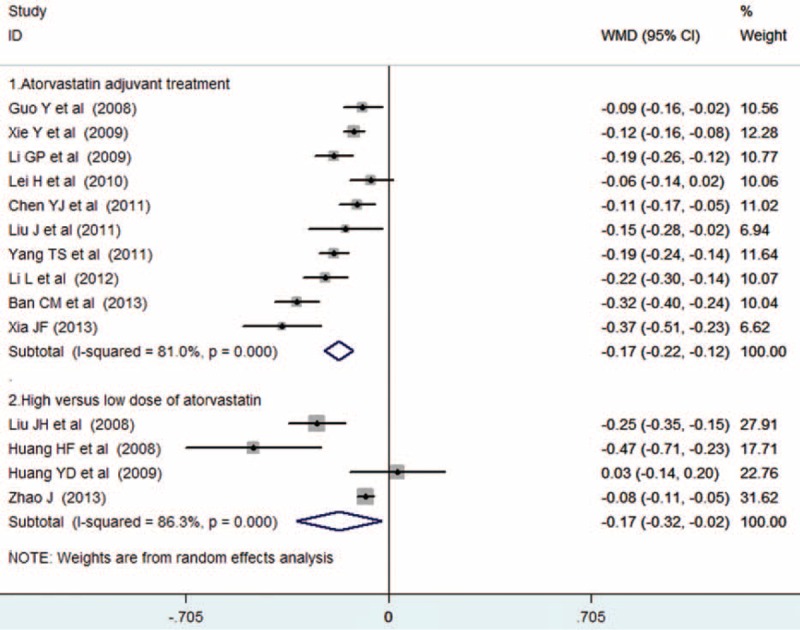
Forest plots showing weighted mean differences with 95% confidence intervals for reduction in carotid intima–media thickness in a random effects model.

**FIGURE 4:**
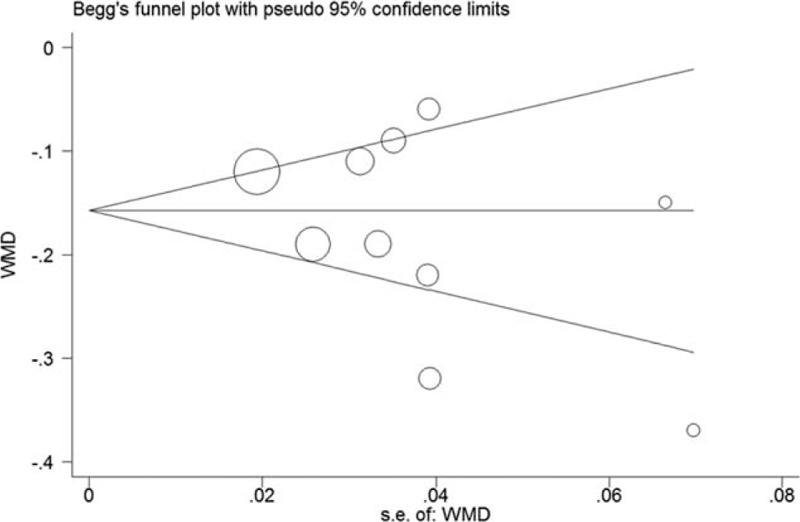
Funnel plots based on the changes of carotid intima–media thickness.

Ten trials^14–23^ provided the add-on effect of atorvastatin treatment on CIMT changes. Of 1025 patients, 524 were allocated to the atorvastatin group, whereas 501 were allocated to the control group. As shown in Figure 3(1), evidence of significant heterogeneity was found (*I*^*2*^ = 81.0%, *P* < 0.001), so we chose the random effects model. Adjuvant treatment with atorvastatin was associated with a significant reduction in CIMT (WMD = −0.17, 95% CI: −0.22 to−0.12). No evidences of publication bias were observed according to Begg's rank correlation test (*P* = 0.283), Egger's linear regression test (*P* = 0.233), and funnel plots (Fig. 4.)

Four trials^24–27^ provided the comparison of high-dose versus low-dose atorvastatin on CIMT changes. Of 320 patients, 163 were allocated to the high-dose atorvastatin group, whereas 157 were allocated to the low-dose group. As shown in Figure 3(2), strong evidence of heterogeneity was also observed (*I*^*2*^ = 86.3%, *P* < 0.001), so we chose the random effects model. High-dose atorvastatin treatment was associated with a significant decrease in CIMT (WMD = −0.17, 95% CI: −0.32 to −0.02). Publication bias was not observed by Begg's rank correlation test (*P* = 0.350) and Egger's linear regression test (*P* = 0.203).

### Changes of Metabolic Parameters

Table 2 lists the changes of total cholesterol (TC), triglyceride (TG), high-density lipoprotein (HDL), low-density lipoproteins (LDL), fasting blood glucose (FBG), glycosylated hemoglobin (HA1c), and postprandial plasma glucose (PPG) between the 2 groups. Overall, adjuvant treatment with atorvastatin was associated with a significant increase in TC (WMD = −1.19 mmol/L, 5% CI −1.79 to −0.60), TG (WMD =  0.53 mmol/L, 95% CI: −0.95 to −0.12), and LDL (WMD = −0.64 mmol/L, 95% CI: −1.16 to −0.12) and increase in HDL-C (WMD = 0.10 mmol/L, 95% CI: 0.00 to 0.20). There were no significant differences in the changes of FPG, PPG, and HA1c between the 2 groups. Compared with the low-dose atorvastatin, high-dose atorvastatin showed some trends in improvement in TC, TG, HDL, LDL, FBG, PPG, and HA1c; however, there were no significant differences in the changes of metabolic parameters between the 2 groups.

**TABLE 2:**
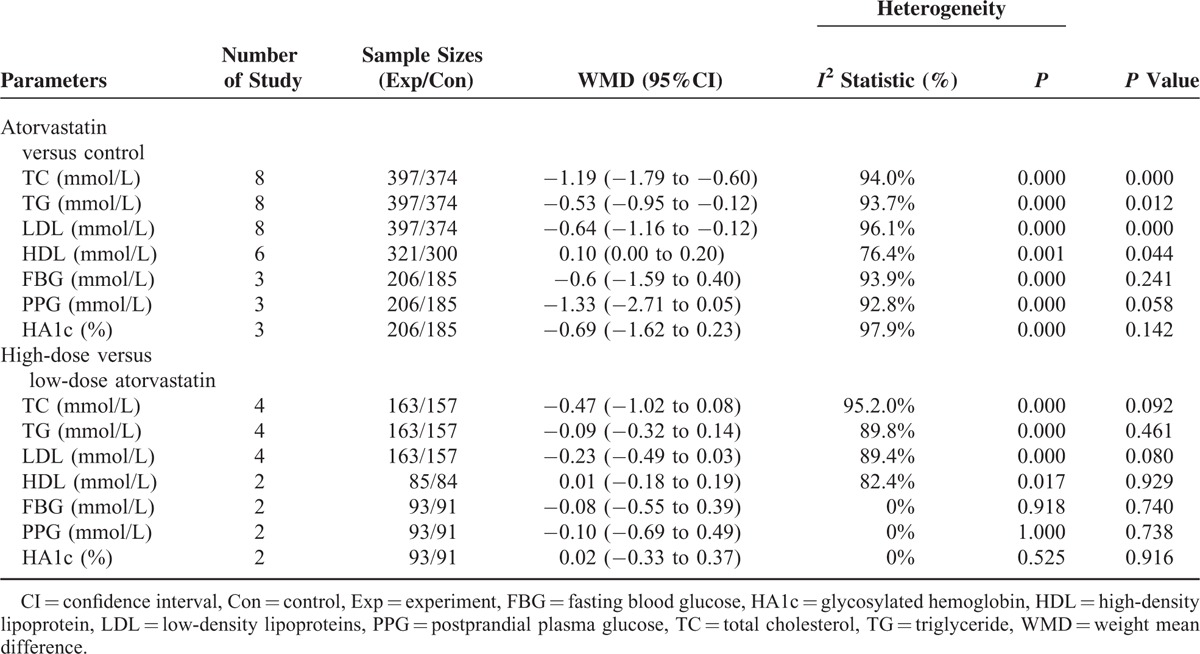
Comparison of the Change of Metabolic Parameters

### Change of High Sensitivity C-reactive protein (hs-CRP)

**FIGURE 5:**
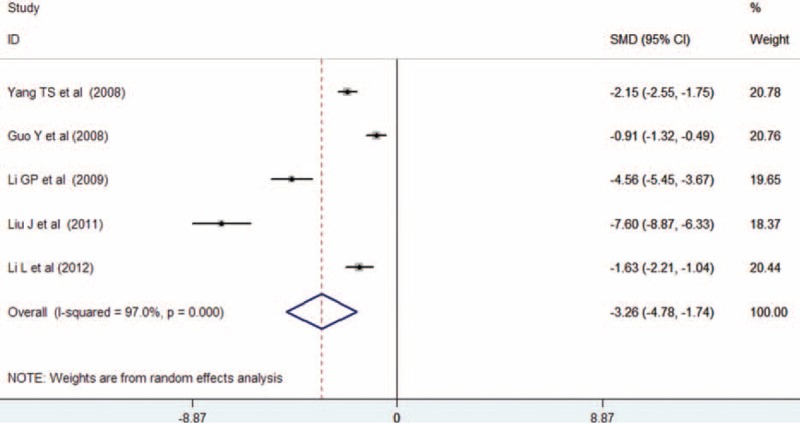
Forest plots showing standardized mean difference with 95% confidence intervals for improvement in serum high sensitivity C-reactive protein levels comparing atorvastatin to the control in a random effects model.

Changes of serum hs-CRP levels were reported in 5 trials.^14,15,17,20,21^ Of 466 patients, 236 were allocated to the high-dose atorvastatin group, whereas 230 were allocated to the low-dose group. As shown in Figure 5, adjuvant treatment with atorvastatin was associated with a significant decrease in hs-CRP (SMD −3.26 mg/L, 95% CI: −4.78 to −1.74) in a random effects model. Evidence of significant heterogeneity was found (*I*^*2*^ = 97%, *P* < 0.001).

### Subgroup Analyses and Sensitivity Analyses

**FIGURE 6:**
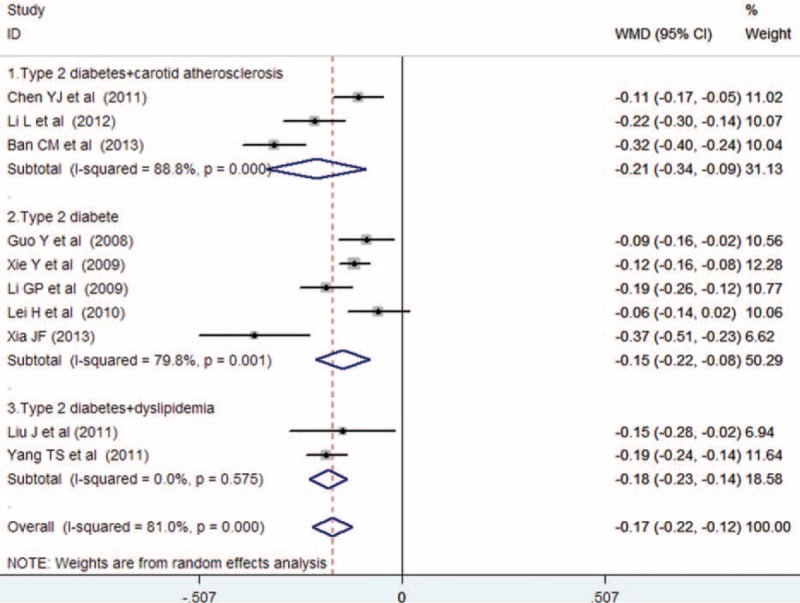
Subgroup analyses of carotid intima-media thickness changes based on the presence of carotid atherosclerosis or dyslipidemia.

Subgroup analyses were conducted based on the changes of CIMT by the presence of complication (carotid atherosclerosis or dyslipidemia). As shown in Figure 6, adjuvant treatment with atorvastatin reduced WMD of CIMT to −0.21 mm (95% CI: −0.22 to −0.12) among patients complicated with carotid atherosclerosis, −0.18 mm (95% CI: −0.23 to −0.14) among patients complicated with dyslipidemia, and −0.15 mm (95% CI: −0.22 to −0.08) among type 2 diabetic patients. Sensitivity analyses were performed by omitting 1 study at each turn to investigate the change of the overall WMD and 95%CI of CIMT. The results showed that there was just a slight change in the WMD or 95% CI, and no change in the direction of WMD when anyone study was omitted (Data not shown).

## DISCUSSION

The main finding of the study is that treatment with atorvastatin significantly reduces CIMT in Chinese patients with type 2 diabetes. High-dose atorvastatin appears to have an additional benefit on the progression of CIMT than the low-dose one. In addition, atorvastatin treatment is associated with reduction in serum hs-CRP levels as well as improvement in serum lipid levels. The beneficial effects of atorvastatin on CIMT regression or slowed progression might be attributable in part to the improvement in lipid profile as well as anti-inflammatory properties. However, atorvastatin treatment has no impact on serum FBG, HA1c, and PPG levels.

The efficacy of atorvastatin on the progression CIMT has been well established.^8^ Although high-dose atorvastatin produced favorable effects on lipid profiles in type 2 diabetes,^28,29^ the impact of atorvastatin on CIMT in patients with type 2 diabetes remains controversial. A study conducted in Greece showed that treatment 10 to 80 mg atorvastatin for 12 months significantly improved the lipid profile but without change of CIMT in type 2 diabetic patients.^9^ A recent report in China found that atorvastatin effectively reduced CIMT in new-onset type 2 diabetes patients.^30^ The relatively short duration of atorvastatin use might be a possible explanation for the conflicting results on CIMT progression.

To our best knowledge, this is the first meta-analysis to investigate atorvastatin on CIMT in type 2 diabetic patients. A systematic review revealed that type 2 diabetes was associated with a 0.13 mm increase in CIMT than the controls.^31^ CIMT, as measured by B-mode ultrasound, is an index of atherosclerotic progression. In the present study, adjuvant treatment with atorvastatin significantly decreased CIMT (WMD = −0.17 mm). Additionally, high-dose atorvastatin treatment appeared to produce greater reduction in CIMT (WMD = −0.17 mm). This result is in agreement with patients treated with high-dose of atorvastatin considerably suppressed CIMT after 12 months than the low-dose atorvastatin ^9^. Subgroup analysis suggested that adjuvant treatment with atorvastatin resulted in a greater reduction in CIMT (WMD = −0.21 mm) among patients complicated with carotid atherosclerosis, suggesting that atorvastatin had beneficial effect on CIMT regression. Moreover, atorvastatin reduced 0.18 mm CIMT in patients complicated with dyslipidemia and 0.15 mm in type 2 diabetic patients. These findings indicated that atorvastatin also had a beneficial effect on slowed CIMT progression. However, all the patients were Chinese people, and generalization of these findings to diverse populations should be cautioned.

Patients with type 2 diabetes typically present with a dyslipidemic profile. Dyslipidaemia in diabetes is mainly characterized by raised triglycerides and reduced HDL cholesterol levels.^32^ Atorvastatin is an effective and safe treatment for hyperlipidemia in Taiwanese diabetic patients.^33^ In the present study, we found that adjuvant treatment with atorvastatin was associated with a significant decrease in serum levels of TC, TG, and LDL levels as well as serum hs-CRP levels. Hs-CRP was a cardiovascular risk predictor in type 2 diabetics with normal lipid profile.^34^These findings suggested that the beneficial effects of atorvastatin on CIMT regression or slowed progression might be attributed to improve the dyslipidemic profile and serum hs-CRP levels.

Increased new-onset insulin resistance and type 2 diabetes risk have raised concerns regarding the use of statin.^35^ Most studies which reported an increased risk of diabetes were conducted in older patients in whom statins were started very late in life.^36,37^ However, drug-specific effects of statins on diabetes risk remain inconclusive. A well-designed meta-analysis suggested that it was important to balance the risks and benefits when administering specific statins.^38^ Animal study showed atorvastatin could prevent the development of type 2 diabetes in the rat model.^39^ Therefore, the risk of new-onset type 2 diabetes in relationship with atorvastatin treatment needs to be long-term follow-up.

Some limitations of this meta-analysis should be noted. First, the relatively low quality of the individual trials reduced the evidence. Second, high heterogeneity (*I*^2^ from 81% and 86.3%) was observed in the analysis of CIMT changes. The most likely sources of heterogeneity might be correlated with the duration of diabetes, dose of atorvastatin treatment, variation in the ultrasound method, and different duration of atorvastatin regimen. Third, blood pressure itself and antihypertensive agents may affect diversely on the results of the CIMT^40,41^; however, the information on blood pressure or antihypertensive agents uses was unavailable in most of the included trials. Finally, despite no evidence of the publication bias on CTIM changes was observed according to Begg's rank correlation test, Egger's linear regression test, and funnel plot, potential publication bias cannot be excluded due to all the included studies were conducted in China and published in Chinese.

## CONCLUSIONS

This meta-analysis suggests that treatment with atorvastatin significantly reduce CIMT in Chinese patients with type 2 diabetes, and particularly contributes to the regression or slowed progression in those complicated with carotid atherosclerosis or dyslipidemia patients. Moreover, high-dose atorvastatin appears to have additional benefits in regression of CIMT than the low-dose atorvastatin. However, due to the methodological drawbacks, more well-designed RCTs are warranted to confirm our findings. In addition, long-term follow-up studies are needed to investigate potential adverse effects following atorvastatin treatment.
